# Protein Source, Dietary Fibre Intake, and Inflammation in Older Adults: A UK Biobank Study

**DOI:** 10.3390/nu17091454

**Published:** 2025-04-26

**Authors:** Mahek Jain, Carlos Celis-Morales, Susan E. Ozanne, Sorrel Burden, Stuart R. Gray, Douglas J. Morrison

**Affiliations:** 1 Scottish Universities Environmental Research Centre (SUERC), University of Glasgow, Glasgow G75 0QF, UK; mahek.jain@glasgow.ac.uk; 2 Human Performance Lab, Education, Physical Activity and Health Research Unit, Universidad Católica del Maule, Talca 34809112, Chile; carlos.celis@glasgow.ac.uk; 3 High-Altitude Medicine Research Centre (CEIMA), Universidad Arturo Prat, Iquique 1101214, Chile; 4 School of Cardiovascular and Metabolic Health, University of Glasgow, Glasgow G12 8TA, UK; stuart.gray@glasgow.ac.uk; 5 Metabolic Research Laboratories and MRC Metabolic Diseases Unit, Institute of Metabolic Science, University of Cambridge, Addenbrookes Hospital Cambridge, Cambridge CB2 0QQ, UK; seo10@cam.ac.uk; 6 Salford Care Organisation, Northern Care Alliance NHS Trust, Stott Lane, Salford M6 8HD, UK; sorrel.burden@manchester.ac.uk; 7 School of Health Sciences, University of Manchester, Oxford Road, Manchester M13 9PL, UK; 8 Institute of Sports Science and Innovation, Lithuanian Sports University, LT-44221 Kaunas, Lithuania

**Keywords:** ageing, protein sources, CRP

## Abstract

Background: Chronic inflammation is linked to cardiovascular disease, cancer, and other non-communicable diseases. Dietary factors like fibre and protein may affect inflammation, but limited evidence exists exploring how they interact. This study investigated associations between dietary fibre, protein sources, and the inflammatory marker *C*-reactive protein (CRP) in older adults. Methods: This cross-sectional analysis included 128,612 UK Biobank participants aged 60+ years with CRP measurements and dietary data from multiple 24 h recalls. Fibre intake was reported as total fibre (g/day). Protein intake included total, animal, and vegetable protein (g/day). Robust regression analysis examined associations between quintiles of fibre, protein, and CRP, adjusted for demographics, lifestyle factors, and multimorbidity. Analyses were stratified by health status (with and without multimorbidity). Results: Higher fibre and vegetable protein intakes were inversely associated with CRP, while higher animal and total protein were positively associated with CRP in people with no multimorbidity. Specifically, participants in the highest quartile of dietary fibre had CRP levels that were 0.42 mg/L lower compared with the lowest quartiles. In contrast, those with the highest total protein and animal protein intakes had CRP levels that were 0.24 mg/L and 0.40 mg/L higher, respectively. In people with multimorbidity, fibre exhibited an inverted U-shaped association with the strongest association in participants in the highest quintile of intake. Vegetable protein had an inverse association with CRP. Animal and total protein had strong positive linear associations with CRP. Notably, high animal protein coupled with low dietary fibre intake resulted in CRP levels that were 0.65 mg/L higher compared with low animal protein and high dietary fibre intake. Conclusions: Higher fibre and vegetable protein intakes were associated with lower inflammation in older adults. In promoting protein intake to maintain muscle mass and function, future studies should investigate replacing animal with vegetable protein to concomitantly reduce age-related inflammation.

## 1. Introduction

Ageing is accompanied by a decline in physical and mental capacity and function which can result in poorer health and wellbeing [1]. In the UK as of 2021, almost 20% of Scotland’s [2] and 18.6% England’s population was above 65 years of age [3]. Ageing is also associated with changes in the innate and adaptive immune system where immunosenence and inflammation can play keys roles in ageing-related morbidity [4]. There is increasing evidence that a key marker of inflammatory status—*C*-reactive protein (CRP)—is not just an important risk factor for inflammation but is also associated with ageing-related morbidity [5]. Moreover, there is robust evidence that CRP is linked to several chronic diseases which are leading causes of mortality worldwide, like cardiovascular disease, chronic kidney disease, cancer, and diabetes mellitus [6]. Evidence from animal models suggest that diet plays an important role in driving host inflammatory responses via changes in the composition and metabolic activity of the gut microbiome, which can affect intestinal permeability and potentially lead to increased pro-inflammatory stimulus from the translocation of gut bacteria and bacterial remnants [7]. However, at the present time and partly because of the inaccessibility to sampling different regions of the gut, there is limited understanding of the complicated interplay between nutrition, the gut microbiota, and chronic systemic inflammation.

CRP is mainly produced by the liver as a part of the acute phase response because of homeostatic shocks like tissue injury or infection. As part of the acute phase response, CRP levels rise exponentially, hence its role as a robust and clinically acceptable biomarker for measuring whole-body inflammation. One of the strongest predictors of CRP levels is adiposity, which indicates that factors influencing adipose cell accretion such as diet could be a potential target to lower levels of CRP [8,9]. Moreover, high CRP levels have been linked to various diseases like cancer [10], diabetes [11], metabolic syndrome, chronic kidney disease, and coronary heart disease [12]. Apart from dietary fibre, multiple dietary nutrients have been reported to influence serum inflammatory markers. The Framingham Heart Offspring cohort study proposed that dietary protein, especially from plant-based sources, was linked to lower levels of inflammatory markers amongst older adults [13]. Moreover, a trend for lower CRP levels was seen in participants with renal diseases when they consumed plant proteins in comparison to those consuming animal proteins (e.g., red meat, eggs) [14].

In addition, large cohort studies have consistently linked high fibre intake (>27 g/day) with a reduction in coronary heart disease risk [15,16]. Dietary fibre escapes digestion in the small intestine but is fermented by the gut microbiota to produce a plethora of small molecules, predominantly short chain fatty acids (SCFAs)—principally acetate, propionate, and butyrate [17]. SCFAs play an important role in maintaining gut epithelial integrity [18] and have pleiotropic effects in shaping the immune system [19]. Through these mechanisms, SCFAs help reduce inflammatory burden by preventing the translocation of pro-inflammatory molecules such as LPS from the gut [20] and promoting overall anti-inflammatory effects [21,22]. In both cross-sectional and longitudinal studies, an inverse relationship is observed between dietary fibre and inflammatory marker levels [23,24]. Ma et al. (2006) demonstrated that, in comparison to subjects in the lower quartile of total fibre intake, participants in the highest quartile had a 63% lower chance of elevated CRP concentrations independent of adiposity levels (OR 0.37, 95% CI; 0.16, 0.87) [25]. Therefore, dietary fibre may have a modulatory role in the interplay between diet, inflammation, and chronic diseases.

As both dietary fibre and protein intake appear important in linking nutrition, inflammation, and chronic diseases, studies looking at the interaction of these dietary factors and its associations with inflammation are needed. There is currently limited evidence available regarding the association of dietary fibre and protein with inflammation. Most of the current evidence base assesses these dietary factors in isolation which could obscure the true nature of the association between protein, fibre, and inflammation [13,26]. Therefore, this study used a large British population from the UK Biobank study to investigate the associations of fibre and different sources of protein intake with CRP levels in older adults.

## 2. Materials and Methods

UK Biobank is a prospective cohort with over 500,000 participants aged between 37 and 73 years from the general UK population (5.5% response rate) [27]. The participants visited 1 of the 22 assessment centres located around England, Scotland, and Wales between 2006 and 2010 [28,29]. In their initial visits, they completed a questionnaire on a touch-screen device, provided samples of their blood, saliva, and urine, and underwent physical measurements. More information about the UK Biobank protocol can be found online (http://www.ukbiobank.ac.uk).

### 2.1. Inclusion Criteria

For the purpose of this study, we included participants equal to or over 60 years of age. The participants were divided into two different subsets of participants: (1) individuals without multimorbidity (free of chronic diseases, including a list of 43 conditions) (https://doi.org/10.1186/s12916-019-1305-x) and (2) individuals who reported having two or more chronic conditions, also known as multimorbidity.

### 2.2. Diet

Dietary intake was measured using the Oxford WebQ, a web-based 24 h dietary assessment tool which collects information on 206 foods and 32 beverages consumed during the 24 h prior to assessment [30]. Furthermore, McCance and Widdowson’s food composition tables were used to calculate the energy and nutrient intake [31]. The dietary assessment tool collects information according to previous day intake via questions like “how much of the following did you drink yesterday” or “did you have any of these yesterday?” For the purpose of this study, an average of five 24 h recalls were used and collected between April 2009 to June 2012.

### 2.3. Dietary Protein Intake

For the purpose of this study, protein intake was categorised by two different sources, animal protein and vegetable protein intake. The sum of animal and vegetable was used to estimate total protein intake. Protein intake was assessed using a combination of self-reported data and dietary recalls and expressed as grams per day (g/day) intake. Participants were asked to report how often they ate different sources of protein. Vegetable protein sources included tempeh, tofu, beans, lentils, nuts, and seeds. Animal protein sources included red meat (beef, pork, lamb, and venison), poultry (chicken, duck, and turkey), eggs (whole eggs, egg yolks, and egg whites), fish (oily fish, such as salmon, herring; mackerel; and white fish, such as cod, haddock, and plaice) and dairy (milk, yoghurt, and cheese).

### 2.4. Dietary Fibre Intake

For the purpose of this study, dietary fibre intake was calculated based on dietary fibre score which was derived from the specified portions and types of vegetables, fruits, breakfast cereal, and bread which contributes to almost 54–60% of the total fibre intake. Dietary fibre intake was expressed as grams per day (g/day) intake. This approach was chosen as it has been shown to distinguish between people with low and high dietary fibre intakes [32,33].

### 2.5. C-Reactive Protein

High-sensitivity CRP was measured in the UK Biobank cohort using particle enhanced immunoturbidimetric assay on a Beckman Coulter AU5800. Plasma samples derived from baseline blood collection (2006–2010) were stored at −80 °C, with CRP levels analysed 4 to 8 years post-collection [34]. CRP level was expressed as milligrams per litre (mg/L). In the UK Biobank project, the reference range for CRP is 0.03 to 5.0 mg/L. CRP levels less than 1 mg/L were regarded low, values between 1.0 and 3.0 mg/L were considered moderate, and levels greater than 3.0 mg/L were considered high [35].

### 2.6. Covariates

Age at baseline was determined using date of birth and baseline assessment. Sex was self-reported at baseline. Ethnicity was also self-reported and was categorised as White or others. Deprivation index (area-based socioeconomic status) was derived from the postcode of residence, utilising the Townsend score. Smoking status was self-reported and was categorised as never, former, or current smoker. Alcohol status was also self-reported and was categorised as daily or almost daily, 3–4 times per week, 1–2 times per week, 1–3 times per month, special occasions or never. At the first visit, participants had their height (cm) and weight (kg) measured at the centre by a trained professional. Multimorbidity was defined as having 2 or more long term conditions out of 43 conditions (Supplementary Table S1). These conditions were derived from a self-reported questionnaire collected at baseline assessment where participants were required to report illnesses for which they have been medically diagnosed. More information on the measurements can be found on the UK Biobank website (http://www.ukbiobank.ac.uk).

### 2.7. Ethical Approval

The Northwest Multi-Centre Research Ethics Committee (Ref: 11/NW/0382) approved the UK Biobank. The protocol for this study is available online at (http://www.ukbiobank.ac.uk/). This research was carried out under the UK Biobank application number 71392.

### 2.8. Statistical Analyses

Descriptive baseline parameters of each dietary nutrient (dietary fibre, vegetable protein, animal protein, and total protein) are reported as means with standard deviations (SD), whereas categorical variables are presented as frequencies and percentages. Normality of variables was assessed using the Anderson–Darling Test for normality.

Associations between fibre/vegetable/animal/total protein with CRP levels were investigated using robust regression analysis. This type of regression allows one to correct for exposures and outcomes that are not within a normal distribution. Protein intake (total, animal, and vegetable) was used as the exposure of interest. These variables were fitted into the model as quartiles, using the lowest quartile of intake as the reference group. Results were reported as adjusted means and their 95% CI for CRP levels.

The association between combined categories of protein and dietary fibre with CRP levels were also investigated. To derive this variable, tertiles for protein (animal and vegetable) and dietary fibre were used which combined generated nine categories of intake, from low protein and low dietary fibre to high protein and high dietary fibre. Participants with low protein and high dietary fibre were used as reference group. Robust regression was performed to estimate the adjusted mean and beta coefficient within categories. The interaction effect between protein and dietary fibre on CRP levels was tested by adding a multiplicative protein fibre interaction into the model. All models were adjusted for sociodemographic (age, sex, deprivation, and ethnicity) and lifestyle factors (smoking and alcohol status).

The analyses were carried out using Stata 18, and graphs were plotted using GraphPad PRISM 10. A *p*-value of less than 0.05 was deemed statistically significant.

## 3. Results

After excluding participants with missing data for any diet indices or scores, 128,612 participants were included in this study.

The baseline characteristics of the cohort by combined categories of protein and dietary fibre intake in people with multimorbidity are reported in Supplementary Table S2. Compared with high total protein intake and low total dietary fibre (HTP-LDF), those with low total protein intake and high dietary fibre (LTP-HDF) were more likely to be women (66.4% vs. 44.8%). Furthermore, the LTP-HDF group had a lower rate of current smokers (3.5% vs. 8.1%) and higher rates of participants with normal BMI (38.2% vs. 19.6%) compared to the HTP-LDF group. In contrast, the HTP-LDF group had a much higher alcohol intake (21.0 vs. 10.8 units) and rates of obesity (34.1% vs. 18.3%). Though deprivation rates were similar between the groups, the LTP-HDF group did have a slightly higher percentage of participants from lower deprivation areas (31.3% vs. 40.65). Overall, these results demonstrate that the LTP-HDF group had a healthier profile at baseline compared to the HTP-LDF group, particularly with regard to smoking, BMI, and alcohol intake.

A comparison of baseline characteristics for LTP-HDF and HTP-LDF groups in the combined tertiles of dietary fibre and total protein in people without multimorbidity is presented in Supplementary Table S3. The LTP-HDF group had a significantly higher proportion of women compared with the HTP-LDF group (65.2% vs. 39.1%, respectively). Additionally, the LTP-HDF group had markedly lower rates of current smoking (2.8% vs. 10.4%) and significantly higher rates of normal BMI (56.6% vs. 33.7%) when compared with the HTP-LDF group. In contrast, the average alcohol intake was substantially higher in the HTP-LDF group relative to the LTP-HDF group (21.72 units vs. 11.75 units). The HTP-LDF group also exhibited dramatically higher obesity rates compared with the LTP-HDF group (15.6% vs. 6.1%). While deprivation levels were similar between the two groups, the LTP-HDF group had a slightly higher percentage of participants from lower deprivation regions (38.4% vs. 37%). In summary, these results indicate that the LTP-HDF group demonstrated an overall healthier risk profile at baseline versus the HTP-LDF group, specifically with regard to smoking status, BMI classification, and alcohol consumption.

**Figure 1:**
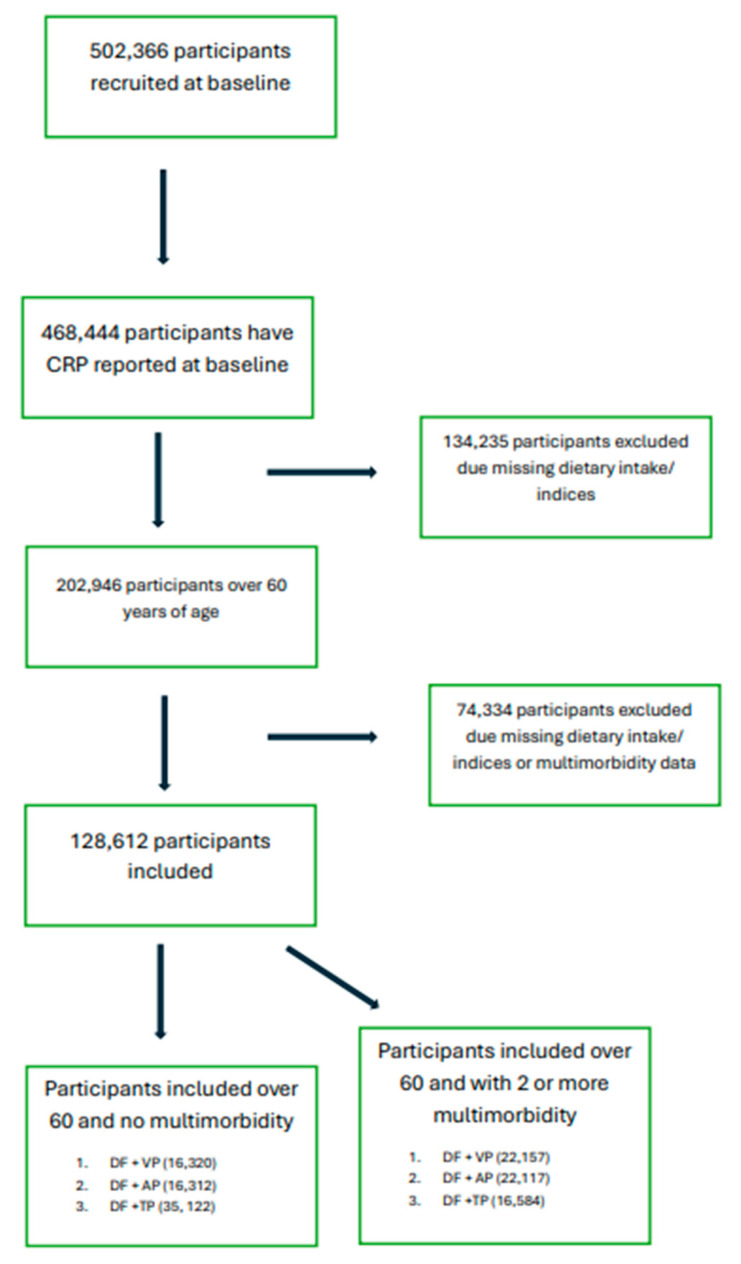
Flow chart for participant selection, UK Biobank.

**Figure 2:**
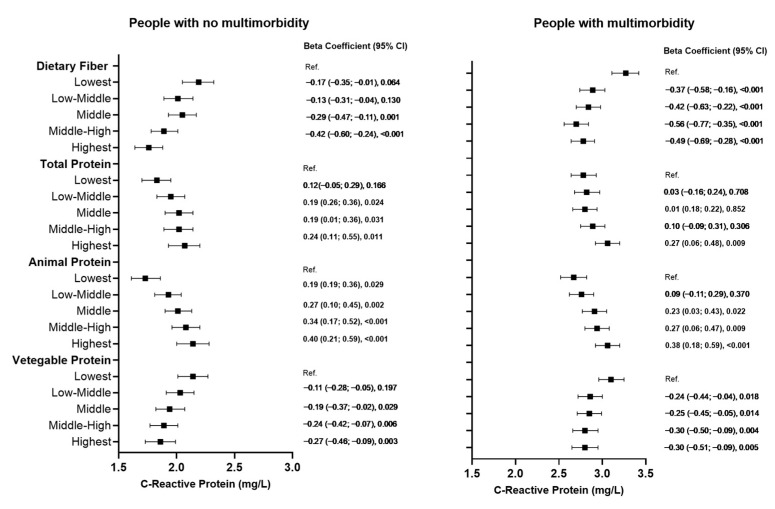
Associations of quintiles of dietary fibre, vegetable, animal and total protein with CRP in participants aged 60 and over. For each comparison, the beta coefficient (95% CI) and significance (*p* value) are stated.

Initial analyses that examined associations between the quintiles of dietary fibre, vegetable, animal, and total protein, and CRP after adjusting for socioeconomic and lifestyle factors are illustrated in Figure 1. Overall, in people without multimorbidity, dietary fibre and vegetable protein had an inverse association with CRP levels. Compared with individuals in the lowest quartile of intake, those in the highest quartile had a −0.42 and −0.27 mg/L lower CRP concentration for dietary fibre and vegetable protein, respectively. Positive associations were observed for total protein and animal protein with CRP concentrations equivalent to 0.24 and 0.40 mg/L higher on those participants with the highest quartile of intake compared with the lowest quartile of intake for total protein and animal protein, respectively (Figure 2). We also examined the association between fibre consumption per 1000 kcal and found that were no substantial differences between quintiles of dietary fibre intake and quintiles of fibre intake per 1000 kcal (Supplementary Figures S1–S3).

**Figure 3:**
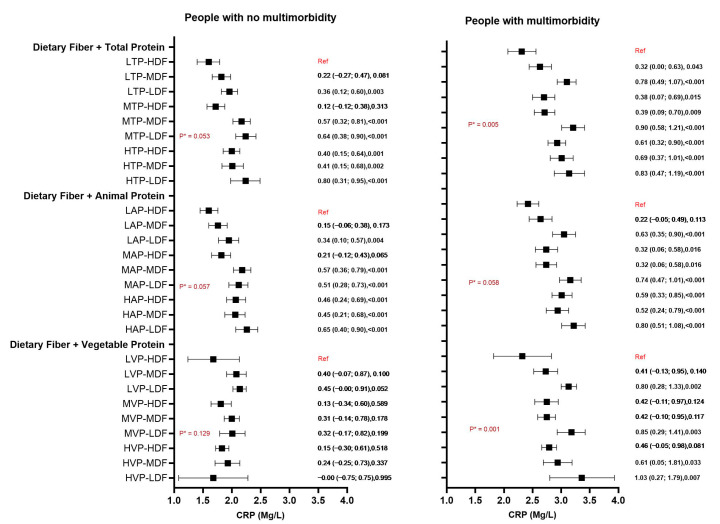
Association of combined tertiles of dietary fibre and protein intake (vegetable, animal, and total) with CRP in older adults with and without multimorbidity. LVP: low vegetable protein, MVP: middle vegetable protein, HVP: high vegetable protein, LAP: low animal protein, MAP: middle animal protein, HAP: high animal protein, LTP: low total protein, MTP: middle total protein, HTP: high total protein, LDF: low dietary fibre, MDF: middle dietary fibre, and HDF: high dietary fibre. For each comparison, the beta coefficient (95% CI) and significance (*p* value) are stated. * indicates significant interaction (*p* < 0.05).

Further, to examine and understand the interaction between protein (vegetable, animal, and total protein) and fibre intake, we categorised these dietary components into tertiles and analysed their combined effects on CRP levels. The association between combined categories of protein and fibre and CRP levels are presented in Figure 2. Among individuals with multimorbidity, significant associations (*p* < 0.001) were found between CRP and both animal and total protein intake combined with dietary fibre intake, but no such associations was observed for vegetable protein (Figure 3). This suggests that the association and interaction observed for total protein is explained by animal protein. Compared with participants consuming low animal protein and high dietary fibre, those with high animal protein but low dietary fibre had the highest levels of CRP, with a 0.65 mg/L difference between these two categories. Overall, among participants with high, middle, and low protein intake, the highest CRP concentrations were always observed for those who also had a low intake of dietary fibre. No association or interaction was observed for vegetable protein and dietary fibre in older adults without multimorbidity.

For adults with multimorbidity, the association between protein and dietary fibre shows a clearer trend and evidence of significant interaction. Higher levels of CRP were observed when the intake of protein increases, and the intake of dietary fibre decreases. For people with multimorbidities, the largest differences were observed for high vegetable protein and the low dietary fibre intake group (CRP + 1.03 mg/L) compared with low vegetable protein and high dietary fibre (Figure 2). Although a similar trend was observed for animal protein and dietary fibre, the magnitude of association was smaller (+0.80 mg/L). We also looked at the association of combined tertiles of vegetable and animal protein with CRP and found that, in people with multimorbidity, higher animal protein intake was associated with higher CRP, irrespective of the amount of vegetable protein consumed (Supplementary Figure S4). Lastly, we conducted sensitivity analyses with a BMI adjustment to examine its impact on our findings, and it was seen that there were minimal differences when compared to our initial models, which were not adjusted for BMI (Supplementary Figure S5).

## 4. Discussion

This study investigated the associations of dietary fibre and protein intake, from various sources, with CRP in people of 60 years of age and above in the UK Biobank cohort. A significant inverse relationship was observed between both dietary fibre and vegetable protein intake with CRP across the intake quintiles in both people with and without multimorbidity. In contrast, animal and total protein exhibited a significant positive association with CRP. The ageing process, with or without age-related disease(s), involves the complex interplay of numerous cellular and molecular signalling pathways. There is increasing evidence linking CRP, as not just an inflammatory biomarker but also as a risk factor associated with ageing-related diseases like hypertension [36], diabetes mellitus [37], cardiovascular diseases [38], and kidney diseases [39]. In recent studies, it has been seen that CRP has been associated with poorer outcomes in several diseases like hypertensive kidney and cardiovascular complications [40], acute and chronic kidney diseases [39], and diabetic neuropathy [41]. Even though, in this study, CRP was measured only at baseline, there is evidence that baseline CRP can help in predicting multimorbidity [42,43]. Biologically, CRP binds to the receptor CD32/CD64 to induce an inflammatory cascade by the activation of NF-κB signalling pathways [5].

In the PREDIMED cohort, older adults in the highest quintile of fibre intake had significantly lower CRP (−1.01; 95% CI: −1.53, −0.49; *p* = 0.004) versus those in the lowest quintile (−0.02; 95% CI: −0.51, 0.48) [44]. Our study is in line with these observations, demonstrating a strong inverse relationship across quintiles of fibre intake in both healthy and unhealthy people. In a US study consisting of older adults, it was seen that a higher fibre intake of 5 g/day was associated with decreased levels of CRP (−0.05; 95% CI: −0.08, −0.01, *p* = 0.007) [45]. The potential mechanisms behind these observations could include changes to the gut microbiota composition, decreased intestinal permeability, and increased SCFA production [46,47].

In a study conducted by Ryoo [48], which assessed the association of dietary protein intake with CRP in older Korean adults with diabetes, it was observed that people who had adequate protein intake had significantly lower CRP in comparison to those who had low protein intake (1.0 ± 1.0 mg/L vs. 1.3 ± 1.6 mg/L). In another cross-sectional study, in 168 older people, CRP was positively associated with an animal-to-vegetable protein ratio in men (r = 0.23, *p* = 0.023) [49]. Moreover, in a meta-analysis of 21 studies encompassing 7365 participants, it was observed that vegan (−0.54 mg/L, 95% CI: −0.79 to −0.28, *p* < 0.0001) and vegetarian diets (−0.25 mg/L, 95% CI: −0.49 to 0.00, *p* = 0.05) were associated with lower levels of CRP in comparison to people who were on a non-vegetarian diet. Specifically, the vegan analysis included 3 studies with 111 vegan participants and 155 non-vegetarian participants, while the vegetarian analysis comprised 14 studies with 2058 vegetarian participants and 5041 non-vegetarian participants [50].

A systematic review of 19 randomised controlled trials demonstrated that higher red meat consumption was associated with elevated CRP levels (0.12 mg/L, 95% CI: 0.04, 0.19). Subgroup analyses revealed that red meat consumption was significantly associated with increased CRP levels in participants with diagnosed diseases (0.20 mg/L, 95% CI: 0.08, 0.32). However, no significant association was observed in participants without diagnosed diseases (−0.04 mg/L (95% CI: −0.17, 0.10) [51]. In a cross-sectional analysis, CRP levels demonstrated significant associations with meat consumption. Each additional 50 g/day of unprocessed red meat intake was associated with a 14.4% increase in CRP (95% CI: 13.6, 15.1%), while total meat consumption showed an 11.6% increase (95% CI: 36.0, 40.7%) [52]. Moreover, in a meta-analysis by Neuenschwander et al., it was observed that replacing red meat with whole grains (0.96; 95% CI: 0.95, 0.98, *n* = 3) and nuts (0.093; 95% CI: 0.91, 0.95, *n* = 9) was linked with decreased risk of all-cause mortality [53]. The results of the present study are in line with past findings, indicating that higher consumption of animal protein including red meat could lead to an increase in CRP levels especially in people with multimorbidity.

In contrast to animal protein, plant protein has a protective effect against elevated CRP, especially in older adults with multimorbidity. Even though animal protein is high-quality protein and an excellent source of indispensable amino acids and micronutrients such as vitamins, iron, and zinc, all of which have several health benefits, animal protein, especially red meat, is associated with higher intakes of saturated fat, salt, cholesterol, iron, phosphate, and iron. Moreover, the elevated consumption of animal protein results in oxidative stress/inflammation, protein/amino acid load, and increased levels of byproducts of protein or AA breakdown by the gut bacteria, such as trimethylamine *n*-oxide or indoxyl sulphate, which may be linked to an elevated risk of cardiovascular disease mortality [54]. While plant proteins have a lower digestibility and amino acid profiles in comparison to animal proteins, recent research suggests that well-planned plant-based diets can meet the protein requirements when consumed in adequate amounts and from diverse sources [55,56]. In contrast, the amino acid content and antioxidant properties of the plant protein likely confer benefits. Moreover, the amino acid and phytochemical content present in the plant proteins are associated with shifts in the gut microbiota which offer anti-inflammatory benefits in humans [57]. Plant proteins derived from, for example, legumes are also rich in fibre which support a healthy gut microbiome, support gut barrier function, and may be important in reducing inflammation [58].

Even though, in our study, we found no strong evidence for synergistic associations between vegetable protein and dietary fibre intakes on CRP, the results indicate the presence of higher systemic inflammation in those with lower intakes of vegetable protein and dietary fibre. The current existing evidence investigates dietary fibre and protein in isolation rather than potential synergistic effects. Moreover, there is a potential research gap in the literature in relation to human studies and the complicated interplay between specific systematic inflammation, nutrients, gut microbiota, and composition.

### Strengths and Limitations

This study had several strengths, with one of them being that we leveraged a large, well-characterised cohort of older British adults to examine the associations between dietary factors and inflammation. The sample size of the UK Biobank provided us with substantial statistical power to detect the differences across intake quintiles. Moreover, the data for various cofounders enabled the adjustment for lifestyle, socioeconomic, and health factors. Lastly, the use of multiple 24 h dietary recalls accounted for intra-individual variability and helped strengthen diet assessments.

However, this study also has several limitations. The reliance on self-reported dietary intake is subject to reporting bias and both underreporting and overreporting, which could lead to inaccuracies in estimating absolute intakes. However, since the 24 h dietary recalls were repeated on multiple occasions, the bias and reporting inaccuracies may have been reduced. Secondly, the study population consisted of primarily White British adults, which limits the generalizability of the results to the wider population. As a predominantly healthy volunteer cohort, UK Biobank participants may not represent the general older adult population. Lastly, since CRP was only measured at baseline, it may not be a true proxy for ongoing systematic inflammation; however, there is evidence which supports the predictive value of baseline CRP in predicting multimorbidity and related health outcomes. Replication in other large cohorts is warranted. Further research through prospective cohort studies and randomised trials should examine these diet–inflammation links over time and test whether purposefully modifying protein and dietary fibre intakes impacts inflammatory outcomes.

## 5. Conclusions

In conclusion, this cross-sectional analysis of older adults in the UK Biobank cohort revealed that higher intakes of dietary fibre and vegetable protein were associated with lower levels of the inflammatory marker CRP. In contrast, higher intakes of animal protein and total protein were associated with higher CRP levels. These associations were more pronounced in participants who had multimorbidities, where fibre showed a negative correlation (Supplementary Figures S1 and S2), and the highest levels of animal and total protein had the strongest positive correlations with CRP. This suggests that, in populations with multimorbidity, moderate fibre intakes and lower animal protein may help reduce systemic inflammation. This study adds to the limited evidence on the interplay between dietary fibre, protein sources, gut microbiota, and inflammation. Future research should examine these associations prospectively and explore potential synergies through dietary pattern analysis. Intervention trials modifying fibre and protein intakes are also needed to determine optimal amounts and ratios for inflammation reduction.

## Data Availability

The data for the findings of this study are available at UK Biobank (http://www.ukbiobank.ac.uk/about-biobank-uk). Restrictions may apply to the availability of these data, which were under licence for the current study (Project ID: 71392).

## Supplementary Materials

The following supporting information can be downloaded at https://www.mdpi.com/article/10.3390/nu17091454/s1, Table S1: List of 43 chronic conditions self-reported included within the definition of multimorbidity in UK Biobank; Table S2: Baseline characteristics across the combined tertiles of dietary fibre and total protein in people with multimorbidity; Table S3: Baseline characteristics across the combined tertiles of dietary fibre and total protein in people without multimorbidity; Figure S1: Association between CRP and quintiles of dietary fibre intake (g/day) in participants over 60 years with no multimorbidity: adjusted marginal means with 95% CI (* denotes significant *p*-value); Figure S2: Association between CRP and quintiles of dietary fibre intake (g/day) in participants over 60 with multimorbidity: adjusted marginal means with 95% CI (* denotes significant *p*-value); Figure S3: Association of quintiles of dietary fibre intake (g/day) and dietary fibre per 1000 kcal with participants aged 60 and over; Figure S4: Association of combined tertiles of vegetable and animal protein with CRP in older adults with and without multimorbidity; Figure S5: Association of vegetable and animal protein with CRP in older adults with and without multimorbidity adjusted for sociodemographic (age, sex, deprivation, BMI and ethnicity) and lifestyle factors (smoking and alcohol status); Abbreviations.
