# Supplementary material for: Protein Source, Dietary Fibre Intake, and Inflammation in Older Adults: A UK Biobank Study
Source: Nutrients. 2025 Apr 26;17(9):1454. doi: 10.3390/nu17091454 (PMC12073801; doi:10.3390/nu17091454)
Supplement: Supplementary file 1 [file nutrients-17-01454-s001.zip › nutrients-3564539-supplementary.pdf]

Supplementary Tables

Supplementary Table S1: List of 43 chronic conditions self-reported included within the definition of multimorbidity in UK Biobank.

**Long term condition grouping**	**Conditions included as reported by participants**
1. Alcohol problems	Alcohol dependency<br>Alcoholic liver disease/alcoholic cirrhosis
2. Anorexia or bulimia	Anorexia<br>Bulimia<br>Other eating disorders
3. Anxiety	Anxiety/panic attacks<br>Nervous breakdown<br>Post-traumatic stress disorder<br>Obsessive compulsive disorder<br>Stress<br>Insomnia<br>Psychological/psychiatric problem
4. Asthma	Asthma
5. Atrial Fibrillation	Atrial Fibrillation
6. Bronchiectasis	Bronchiectasis
7. Cancer	Lifetime diagnosis
8. Chronic fatigue syndrome	Chronic fatigue syndrome
9. Chronic kidney disease	Polycystic kidney<br>Diabetic nephropathy<br>Renal/kidney failure

	Renal failure requiring dialysis<br>Renal failure not requiring dialysis<br>Kidney nephropathy<br>Immunoglobulin A (IgA) nephropathy
10. Chronic Liver disease	Oesophageal varices<br>Non infective hepatitis<br>Liver failure/cirrhosis<br>Primary biliary cirrhosis
11. Chronic sinusitis	Chronic sinusitis
12. Chronic Obstructive Pulmonary Disease (COPD)	COPD/chronic obstructive airways disease<br>Emphysema/chronic bronchitis<br>Emphysema
13. Connective tissue disorders	Myositis/myopathy<br>Systemic Lupus Erythematosus<br>Connective tissue disorder<br>Sjogrens syndrome/sicca syndrome<br>Dermatopolymyositis<br>Scleroderma/systemic sclerosis<br>Rheumatoid arthritis<br>Psoriatic arthropathy<br>Dermatomyositis<br>Polymyositis<br>Polymyalgia Rheumatica<br>Malabsorption/coeliac disease

14. Coronary Heart Disease	Heart attack/Myocardial Infarction<br><br>Angina
15. Depression	Depression<br><br>Postnatal Depression
16. Dementia	Dementia<br><br>Alzheimer’s disease<br><br>Cognitive impairment
17. Diabetes	Diabetic nephropathy<br><br>Diabetic neuropathy/ulcers<br><br>Diabetes<br><br>Type 1 diabetes<br><br>Type 2 diabetes<br><br>Diabetic eye disease
18. Diverticular disease	Diverticular disease<br><br>Diverticulitis
19. Dyspepsia	Gastro-oesophageal reflux (GORD)/gastric reflux<br><br>Oesophagitis /Barrett's oesophagus<br><br>Gastric stomach ulcers<br><br>Gastric erosions/gastritis<br><br>Duodenal ulcer<br><br>Dyspepsia/indigestion<br><br>Hiatus hernia<br><br>Helicobacter pylori

20. Endometriosis	Endometriosis
21. Epilepsy	Epilepsy
22. Glaucoma	Glaucoma
23. Heart failure	Cardiomyopathy<br>Hypertrophic cardiomyopathy<br>Heart failure/pulmonary oedema
24. Hypertension	Hypertension<br>Essential Hypertension
25. Inflammatory Bowel Disease	Inflammatory Bowel Disease<br>Crohn's disease<br>Ulcerative colitis
26. Irritable bowel syndrome	Irritable bowel syndrome
27. Meniere's disease	Meniere's disease
28. Migraine	Migraine
29. Multiple Sclerosis	Multiple Sclerosis
30. Osteoporosis	Osteoporosis
31. Other psychoactive substance abuse	Opioid dependency<br>Other substance abuse/dependency
32. Painful conditions	Back pain<br>Joint pain<br>Back pain<br>Joint pain<br>Headaches (not migraine)<br>Sciatica

	Plantar fasciitis<br><br>Carpal tunnel syndrome<br><br>Fibromyalgia<br><br>Arthritis<br><br>Shingles<br><br>Disc problem<br><br>Prolapsed disc/slipped disc<br><br>Spine arthritis/spondylitis<br><br>Ankylosing spondylitis<br><br>Back problem<br><br>Osteoarthritis<br><br>Gout<br><br>Cervical spondylosis<br><br>Trigeminal neuralgia<br><br>Disc degeneration<br><br>Trapped nerve/compressed nerve
33. Parkinson's disease	Parkinson's disease
34. Peripheral vascular disease	Peripheral vascular disease<br><br>Leg claudication/intermittent claudication
35. Pernicious Anaemia	Pernicious Anaemia
36. Polycystic ovary	Polycystic ovary
37. Prostate disorders	Prostate problem (not cancer)<br><br>Enlarged prostate<br><br>Benign prostatic hypertrophy

38. Psoriasis/eczema	Eczema<br>Dermatitis<br>Psoriasis
39. Schizophrenia/bipolar disorder	Schizophrenia<br>Mania/<br>Bipolar disorder<br>Manic depression
40. Stroke/Transient Ischaemic Attack (TIA)	Stroke<br>TIA<br>Subarachnoid haemorrhage<br>Brain haemorrhage<br>Ischaemic stroke
41. Thyroid disorders	Thyroid problem (not cancer)<br>Hyperthyroidism/thyrotoxicosis<br>Hypothyroidism/myxoedema<br>Grave's disease<br>Thyroid goitre<br>Thyroiditis
42. Treated constipation	Constipation
43. Viral Hepatitis	Infective/viral hepatitis<br>Hepatitis B<br>Hepatitis C<br>Hepatitis D<br>Hepatitis E

Supplementary Table S2: Baseline characteristics across the combined tertiles of dietary fibre and total protein in people with multimorbidity.

	**Total**	**LTP-HDF**	**LTP-LDF**	**HTP-HDF**	**HTP-LDF**
N=, (%)	43,861 (100)	3,011 (6.86)	6,839 (15.59)	7,166 (16.34)	3,130 (7.14)
Age (years), mean (SD)	64.5 (2.8)	64.2 (2.8)	64.2 (2.7)	64.3 (2.7)	64.07 (2.7)
Sex, n= (%)					
Women	35,903 (52.2)	1,157 (66.4)	2,613 (70.2)	1,688 (39.6)	660 (44.8)
Men	32,808 (47.7)	583 (33.5)	1,106 (29.7)	2,575 (60.4)	811 (55.1)
Deprivation index, N= (%)					
Lower deprivation	21,506 (31.3)	611 (35.2)	1,303 (35.0)	1,731 (40.6)	515 (35.0)
Middle	22,545 (32.8)	611 (35.2)	1,319 (35.5)	1,469 (34.5)	533 (36.3)
Higher deprivation	24,610 (35.8)	513 (29.5)	1,092 (29.4)	1,057 (24.8)	422(28.7)
Smoking status, N= (%)					
Never	30,751 (45.1)	918 (52.9)	1,712 (46.1)	2,023 (47.7)	576 (39.3)
Previous	30,917 (45.3)	754 (43.4)	1,686 (45.4)	2,046 (48.1)	770 (52.5)
Current	6,466 (9.4)	62 (3.58)	312 (8.4)	174 (4.09)	119 (8.1)
Alcohol Intake (in units), mean (SD)	14.92 (19.3)	10.83 (13.6)	16.56 (19.6)	15.31 (16.9)	21.04 (21.7)
BMI kg/m^2, mean (SD)	29.0 (5.0)	26.4 (4.1)	26.8 (4.1)	28.2 (4.5)	28.7 (4.5)
BMI Categories, n= (%)					
Underweight	250 (0.3)	15 (0.8)	15 (0.4)	9 (0.2)	3 (0.2)
Normal	13,547 (19.8)	663 (38.2)	1,315 (35.6)	1,002 (23.5)	287 (19.6)

Overweight	29,394 (43.0)	738 (42.5)	1,611 (43.7)	1.965 (46.2)	672 (46.0)
Obese	25,103 (36.7)	319 (18.9)	745 (20.2)	1,272 (29.9)	499 (34.1)

Supplementary Table S3: Baseline characteristics across the combined tertiles of dietary fibre and total protein in people without multimorbidity.

	**Total**	**LTP-HDF**	**LTP-LDF**	**HTP-HDF**	**HTP-LDF**
N=, (%)	34,885 (100)	1,458 (100)	2,791 (100)	2,776 (100)	871 (100)
Baseline age (years), mean (SD)	63.8 (2.8)	63.5 (2.6)	63.6 (2.6)	63.53 (2.6)	63.4 (2.6)
Sex, n= (%)					
Women	18,053 (51.7)	951 (65.2)	1,949 (69.8)	1,069 (38.1)	341 (39.1)
Men	16,817 (48.2)	507 (34.7)	842 (30.1)	1,707 (61.4)	530 (60.8)
Deprivation index, N= (%)					
Lower deprivation	13,343 (38.4)	545 (37.4)	1,048 (37.5)	1,108 (39.9)	322 (37.0)
Middle	12,085 (34.6)	540 (37.0)	1,017 (36.4)	1,017 (36.6)	315 (26.2)
Higher deprivation	9,414 (27.0)	372 (25.5)	724 (25.9)	649 (23.4)	233 (26.7)
Smoking status, N= (%)					
Never	18,633 (53.9)	902 (62.1)	1,494 (53.7)	1,577 (56.9)	426 (48.9)
Previous	12,525 (36.2)	508 (35.0)	1,058 (38.0)	1,056 (38.1)	353 (89.5)
Current	3,365 (9.7)	41 (2.8)	228 (8.2)	138 (4.9)	91 (10.4)
Alcohol Intake, mean (SD)	16.1 (18.0)	11.7 (12.2)	17.1 (16.8)	16.2 (15.4)	21.7 (21.1)

BMI kg/m^2, mean (SD)	26.6 (3.89)	24.7 (3.2)	25.1 (3.3)	26.0 (3.6)	26.6 (3.8)
BMI n=, (%)					
Underweight	166 (0.4)	15 (1.0)	18 (0.6)	12 (0.4)	6 (0.6)
Normal	12,098 (35.1)	823 (56.6)	1,419 (51.0)	1,138 (41.1)	293 (33.7)
Overweight	16,283 (47.2)	525 (36.1)	1,136 (40.8)	1,241 (44.8)	434 (49.9)
Obese	5,917 (17.1)	89 (6.1)	208 (7.4)	378 (13.6)	136 (15.6)

Supplementary Figure S1: Association between CRP and quintiles of dietary fibre intake (g/day) in participants over 60 years with no multimorbidity: adjusted marginal means with 95% CI (* denotes significant p-value).

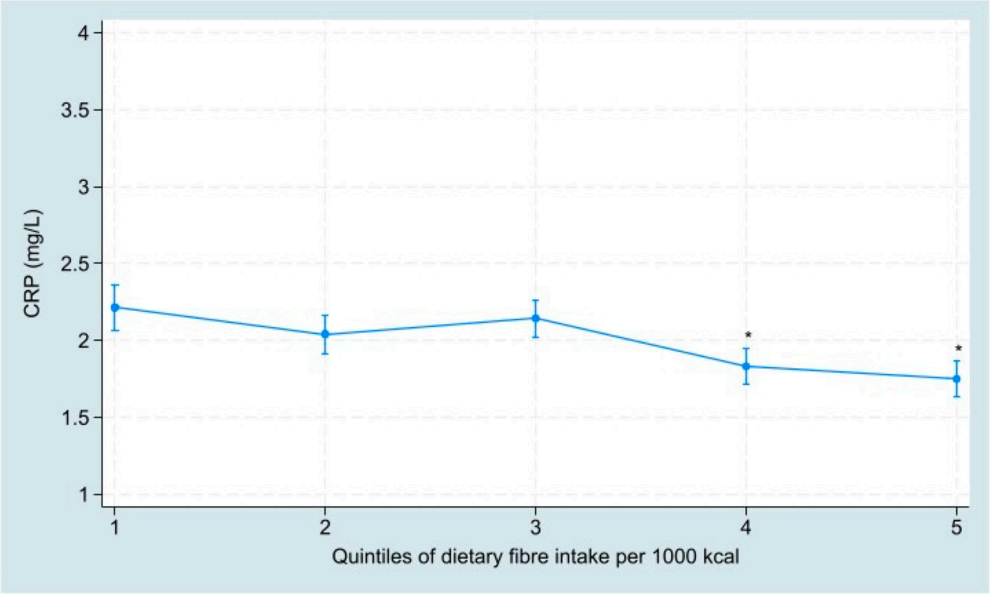

Supplementary Figure S2: Association between CRP and quintiles of dietary fibre intake (g/day) in participants over 60 with multimorbidity: adjusted marginal means with 95% CI (* denotes significant p-value).

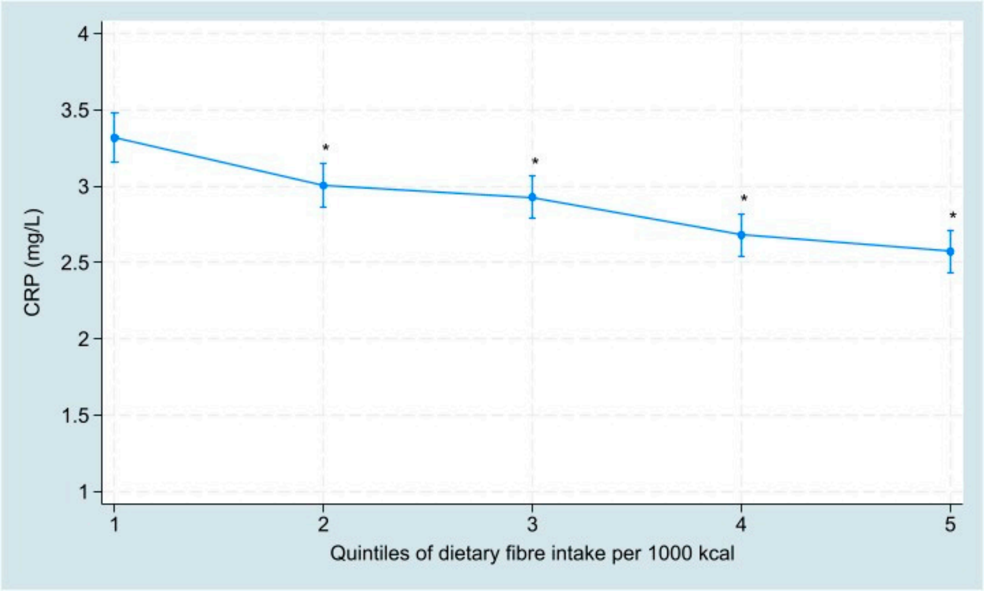

Supplementary Figure S3: Association of quintiles of dietary fibre intake (g/day) and dietary fibre per 1000 kcal with participants aged 60 and over.

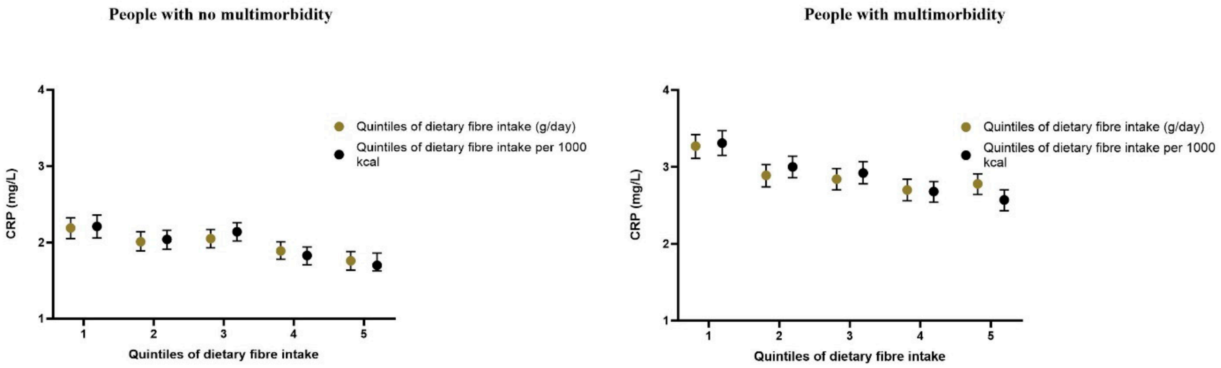

Supplementary Figure S4: Association of combined tertiles of vegetable and animal protein with CRP in older adults with and without multimorbidity.

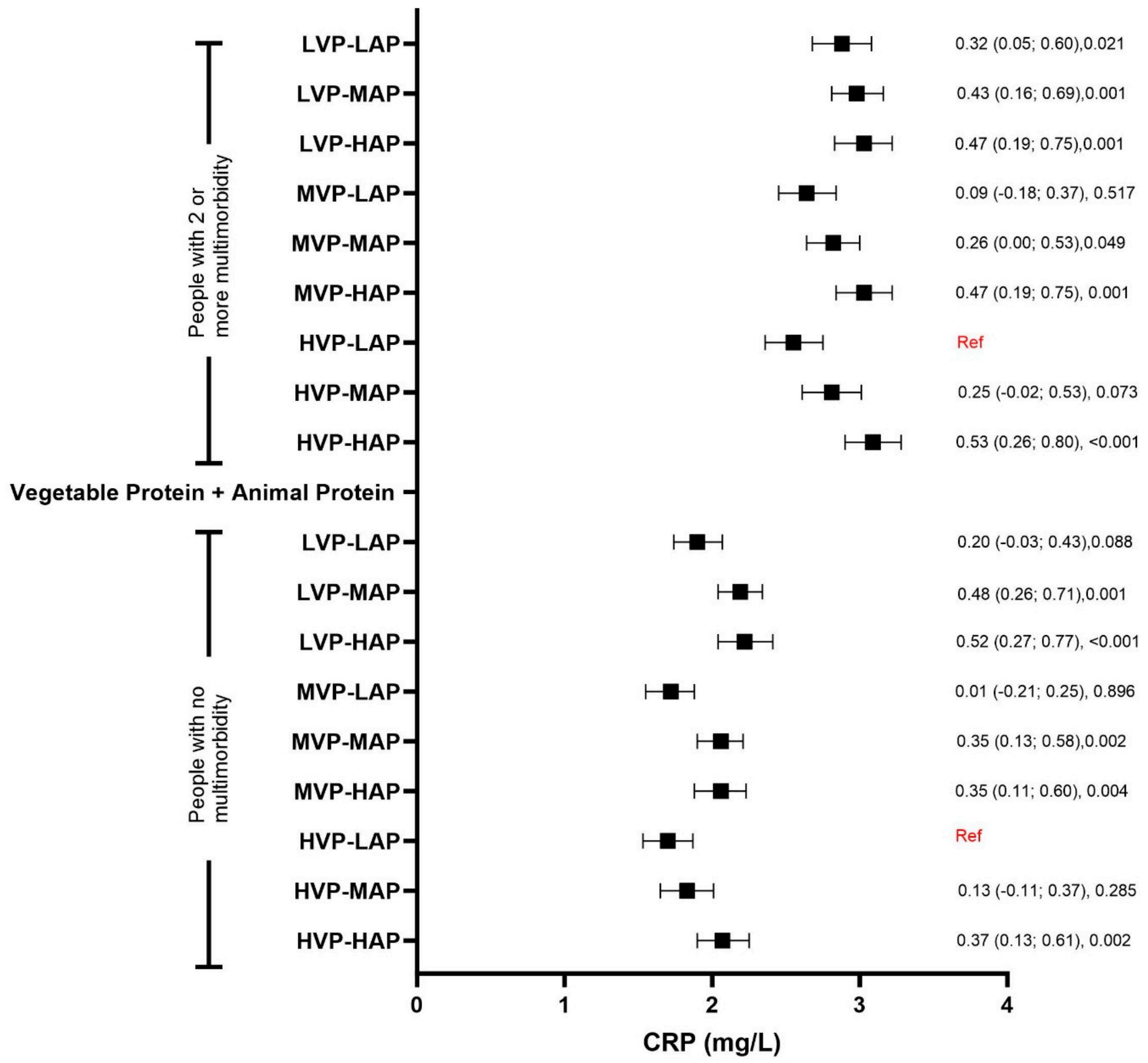

Supplementary Figure S5: Association of vegetable and animal protein with CRP in older adults with and without multimorbidity adjusted for sociodemographic (age, sex, deprivation, BMI and ethnicity) and lifestyle factors (smoking and alcohol status).

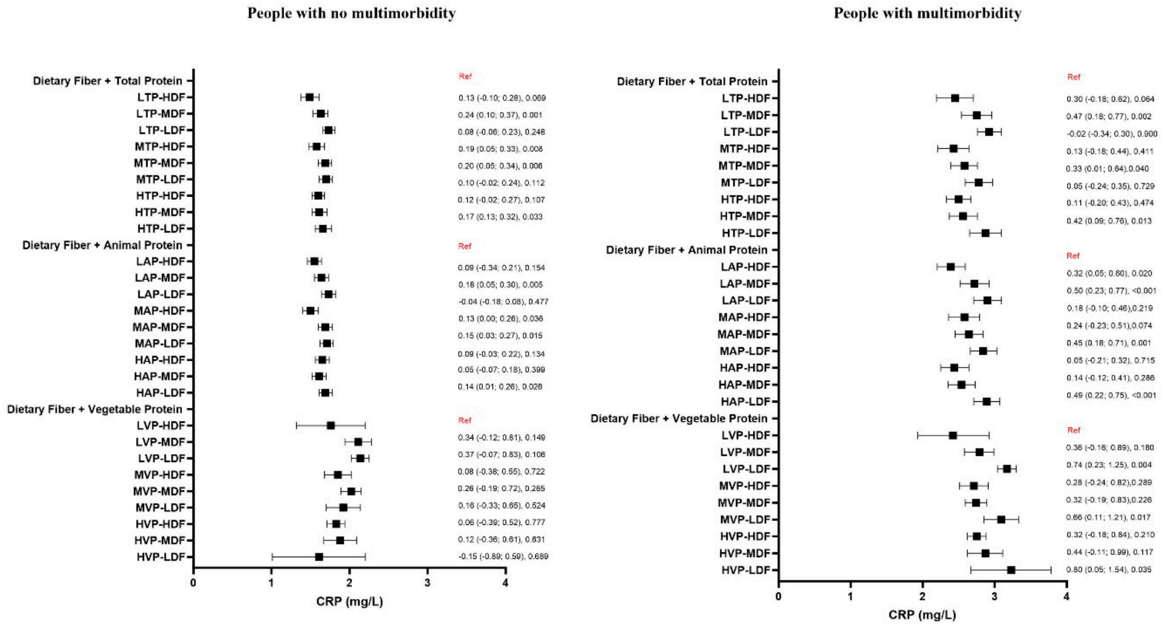

Abbreviations:

- DF – Dietary fibre
- VP – Vegetable Protein
- AP – Animal Protein
- TP – Total Protein
- HTP – High Total Protein
- MTP – Medium Total Protein
- LTP – Low Total Protein
- HVP – High Vegetable Protein
- MVP – Medium Vegetable Protein
- LVP – Low Vegetable Protein
- HAP – High Animal Protein
- MAP – Medium Animal Protein
- LAP – Low Animal Protein
- HDF – High Dietary fibre
- MDF – Medium Dietary fibre
- LDF – Low Dietary fibre
- P* - p value for interaction
- CRP – C – Reactive Protein
- SCFA – Short Chain Fatty Acids
